# 3,6-Dimethyl-1-phenyl-4-(2-thien­yl)-8-(2-thienylmethyl­ene)-5,6,7,8-tetra­hydro-1*H*-pyrazolo[3,4-*b*][1,6]naphthyridine

**DOI:** 10.1107/S1600536809014810

**Published:** 2009-04-25

**Authors:** Juhua Peng, Zhengguo Han, Ning Ma, Shujiang Tu

**Affiliations:** a Lianyungang Teachers’ College, Lianyungang 222006, People’s Republic of China; b College of Chemistry and Chemical Engineering, Xuzhou Normal University, Xuzhou 221116, People’s Republic of China

## Abstract

In the mol­ecule of the title compound, C_26_H_22_N_4_S_2_, the pyrazole ring is oriented at a dihedral angle of 0.85 (3)° with respect to the adjacent naphthyridine ring, while the other ring of naphthyridine adopts an envelope conformation. The dihedral angle between phenyl and pyrazole rings is 87.65 (3)°. In the crystal structure, weak inter­molecular C—H⋯N inter­actions link the mol­ecules into chains. The π–π contacts between the naphthyridine rings and the naphthyridine and thio­phene rings [centroid–centroid distances = 3.766 (3) and 3.878 (3) Å] may further stabilize the structure. A weak C—H⋯π inter­action is also present.

## Related literature

For the biological activity of naphthyridines, see: Abou *et al.* (2001 ▶); Aleem *et al.* (2002 ▶); Blagg *et al.* (2003 ▶); Ohta *et al.* (2004 ▶). For the biological properties of pyrazolopyridine derivatives, see: Lynck *et al.* (1988 ▶); Fucini *et al.* (2008 ▶); Warshakoon *et al.* (2006 ▶). They are also active against gram positive and gram negative bacteria, see: El-Dean *et al.* (1991 ▶) and inhibit cholesterol formation, see: Fujikawa *et al.* (1989 ▶, 1990 ▶). For bond-length data, see: Allen *et al.* (1987 ▶).
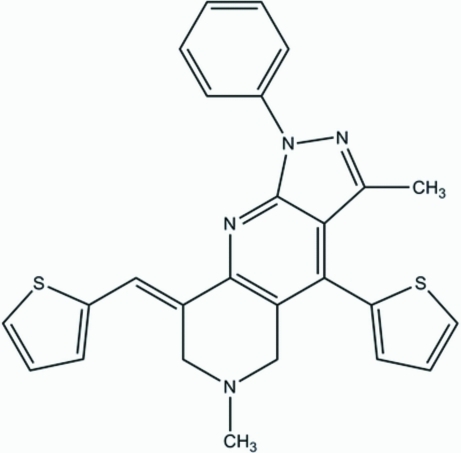


## Experimental

### 

#### Crystal data

C_26_H_22_N_4_S_2_
*M* *_r_* = 454.60
Triclinic, 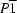

*a* = 10.7187 (16) Å
*b* = 10.9704 (19) Å
*c* = 11.153 (2) Å
α = 109.785 (2)°
β = 102.364 (1)°
γ = 104.201 (1)°
*V* = 1131.0 (3) Å^3^
*Z* = 2
Mo *K*α radiation
μ = 0.26 mm^−1^
*T* = 298 K
0.18 × 0.17 × 0.16 mm

#### Data collection

Bruker SMART CCD area-detector diffractometer
Absorption correction: multi-scan (*SADABS*; Sheldrick, 1996 ▶) *T*_min_ = 0.955, *T*_max_ = 0.960
5846 measured reflections
3918 independent reflections
2322 reflections with *I* > 2σ(*I*)
*R*_int_ = 0.025

#### Refinement

*R*[*F* ^2^ > 2σ(*F* ^2^)] = 0.057
*wR*(*F* ^2^) = 0.176
*S* = 1.00
3918 reflections
291 parameters
H-atom parameters constrained
Δρ_max_ = 0.31 e Å^−3^
Δρ_min_ = −0.43 e Å^−3^

### 

Data collection: *SMART* (Bruker, 1998 ▶); cell refinement: *SAINT* (Bruker, 1998 ▶); data reduction: *SAINT*; program(s) used to solve structure: *SHELXS97* (Sheldrick, 2008 ▶); program(s) used to refine structure: *SHELXL97* (Sheldrick, 2008 ▶); molecular graphics: *ORTEP-3 for Windows* (Farrugia, 1997 ▶) and *PLATON* (Spek, 2009 ▶); software used to prepare material for publication: *SHELXL97*.

## Supplementary Material

Crystal structure: contains datablocks global, I. DOI: 10.1107/S1600536809014810/hk2672sup1.cif

Structure factors: contains datablocks I. DOI: 10.1107/S1600536809014810/hk2672Isup2.hkl

Additional supplementary materials:  crystallographic information; 3D view; checkCIF report

## Figures and Tables

**Table 1:** Hydrogen-bond geometry (Å, °)

*D*—H⋯*A*	*D*—H	H⋯*A*	*D*⋯*A*	*D*—H⋯*A*
C26—H26⋯N2^i^	0.93	2.57	3.445 (3)	157
C20—H20⋯*Cg*6^ii^	0.93	2.93	3.680 (3)	139

Symmetry codes: (i) 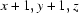; (ii) 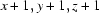. *Cg*6 is the centroid of the S1/C23–C26 ring.

**Fig. 1.**
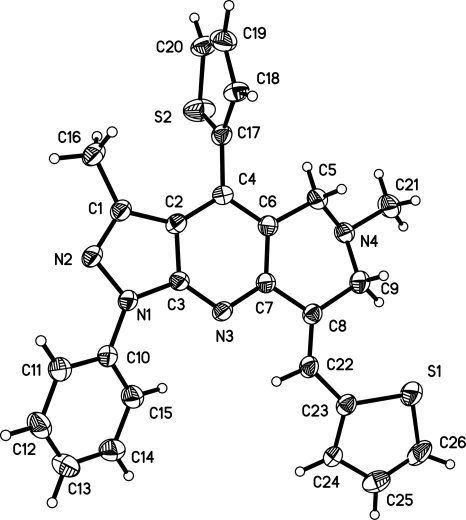
The molecular structure of the title molecule, with the atom-numbering scheme. Displacement ellipsoids are drawn at the 30% probability level.

**Fig. 2.**
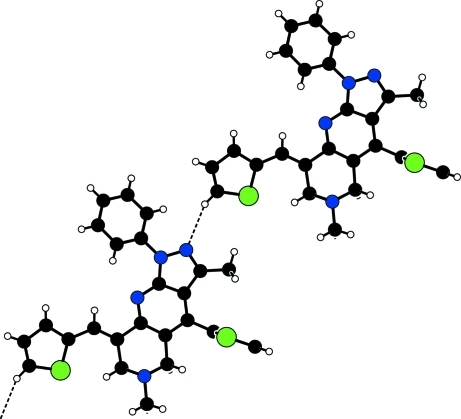
A partial packing diagram of the title compound. Hydrogen bonds are shown as dashed lines.

## supplementary crystallographic information

### Comment

Naphthyridines have received considerable attention over the past years because of their wide range of biological activities including antitumor (Abou *et al.*,  2001; Aleem *et al.*,  2002), anti-inflammatory (Blagg *et al.*,  2003) and antifungal (Ohta *et al.*,  2004) activities. Pyrazolopyridine derivatives are important heterocyclic compounds, which exhibit a diverse range of biological properties such as new inhibitors of xanthine oxidases (Lynck *et al.*, 1988), as Polo-like kinase 1 inhibitors (Fucini *et al.*,  2008) and HIF-1alpha prolyl hydroxylase inhibitors (Warshakoon *et al.*, 2006). They also have proven to be active against gram positive and gram negative bacterias (El-Dean *et al.*, 1991) and also as compounds for the inhibition of cholesterol formation (Fujikawa *et al.*,  1989, 1990). We report herein the crystal structure of the title compound, containing the skeletons of naphthyridine and pyrazolopyridine.

In the molecule of the title compound (Fig. 1), the bond lengths (Allen *et al.*,  1987) and angles are within normal ranges. Rings A (N1/N2/C1-C3), B (N3/C2-C4/C6/C7), D (C10-C15), E (S2/C17-C20) and F (S1/C23-C26) are, of course, planar, and they are oriented at dihedral angles of A/B = 0.85 (3), A/D = 87.65 (3) and B/E = 18.10 (4) °. Ring C (N4/C5-C9) adopts envelope conformation, with atom N4 displaced by 0.660 (3) Å from the plane of the other ring atoms.

In the crystal structure, weak intermolecular C-H···N interactions (Table 1) link the molecules into chains (Fig. 2), in which they may be effective in the stabilization of the structure. The π–π contacts between the naphthyridine rings and the naphthyridine and thiophene rings, Cg2—Cg2^i^ and Cg2—Cg6^ii^ [symmetry codes: (i) 1 - x, 1 - y, 1 - z, (ii) -x, 1 - y, 1 - z, where Cg2 and Cg6 are centroids of the rings B (N3/C2-C4/C6/C7) and F (S1/C23-C26), respectively] may further stabilize the structure, with centroid-centroid distances of 3.766 (3) and 3.878 (3) Å, respectively. There also exists a weak C-H···π interaction (Table 1).

### Experimental

The title compound was prepared by the reaction of of 1-methyl-3,5-bis(thiophen -2-ylmethylene)piperidin-4-one (1 mmol) and 3-methyl-1-phenyl-1*H*-pyrazol -5- amine (1 mmol) in glycol (2 ml).

### Refinement

H atoms were positioned geometrically, with C-H = 0.93, 0.97 and 0.96 Å for aromatic, methylene and methyl H, respectively, and constrained to ride on their parent atoms, with U_iso_(H) = xU_eq_(C), where x = 1.5 for methyl H and x = 1.2 for all other H atoms.

### Crystal data

**Table:**

C_26_H_22_N_4_S_2_	*Z* = 2
*M**_r_* = 454.60	*F*(000) = 476
Triclinic, *P*1	*D*_x_ = 1.335 Mg m^−^^3^
Hall symbol: -P 1	Melting point = 452–453 K
*a* = 10.7187 (16) Å	Mo *K*α radiation, λ = 0.71073 Å
*b* = 10.9704 (19) Å	Cell parameters from 1616 reflections
*c* = 11.153 (2) Å	θ = 2.3–24.8°
α = 109.785 (2)°	µ = 0.26 mm^−^^1^
β = 102.364 (1)°	*T* = 298 K
γ = 104.201 (1)°	Block, yellow
*V* = 1131.0 (3) Å^3^	0.18 × 0.17 × 0.16 mm

### Data collection

**Table:**

Bruker SMART CCD area-detector diffractometer	3918 independent reflections
Radiation source: fine-focus sealed tube	2322 reflections with *I* > 2σ(*I*)
graphite	*R*_int_ = 0.025
φ and ω scans	θ_max_ = 25.0°, θ_min_ = 2.1°
Absorption correction: multi-scan (*SADABS*; Sheldrick, 1996)	*h* = −12→11
*T*_min_ = 0.955, *T*_max_ = 0.960	*k* = −12→13
5846 measured reflections	*l* = −13→13

### Refinement

**Table:**

Refinement on *F*^2^	Primary atom site location: structure-invariant direct methods
Least-squares matrix: full	Secondary atom site location: difference Fourier map
*R*[*F*^2^ > 2σ(*F*^2^)] = 0.057	Hydrogen site location: inferred from neighbouring sites
*wR*(*F*^2^) = 0.176	H-atom parameters constrained
*S* = 1.00	*w* = 1/[σ^2^(*F*_o_^2^) + (0.0957*P*)^2^] where *P* = (*F*_o_^2^ + 2*F*_c_^2^)/3
3918 reflections	(Δ/σ)_max_ < 0.001
291 parameters	Δρ_max_ = 0.31 e Å^−^^3^
0 restraints	Δρ_min_ = −0.42 e Å^−^^3^

### Special details

**Table:**

Geometry. All e.s.d.'s (except the e.s.d. in the dihedral angle between two l.s. planes) are estimated using the full covariance matrix. The cell e.s.d.'s are taken into account individually in the estimation of e.s.d.'s in distances, angles and torsion angles; correlations between e.s.d.'s in cell parameters are only used when they are defined by crystal symmetry. An approximate (isotropic) treatment of cell e.s.d.'s is used for estimating e.s.d.'s involving l.s. planes.
Refinement. Refinement of *F*^2^ against ALL reflections. The weighted *R*-factor *wR* and goodness of fit *S* are based on *F*^2^, conventional *R*-factors *R* are based on *F*, with *F* set to zero for negative *F*^2^. The threshold expression of *F*^2^ > σ(*F*^2^) is used only for calculating *R*-factors(gt) *etc*. and is not relevant to the choice of reflections for refinement. *R*-factors based on *F*^2^ are statistically about twice as large as those based on *F*, and *R*- factors based on ALL data will be even larger.

### Fractional atomic coordinates and isotropic or equivalent isotropic displacement parameters (Å^2^)

**Table:**

	*x*	*y*	*z*	*U*_iso_*/*U*_eq_	
S1	1.09086 (12)	0.85725 (11)	1.54317 (11)	0.0768 (4)	
S2	0.32900 (12)	0.10633 (12)	1.04051 (11)	0.0826 (4)	
N1	0.5616 (3)	0.2524 (3)	1.5917 (3)	0.0481 (7)	
N2	0.4340 (3)	0.1519 (3)	1.5362 (3)	0.0529 (8)	
N3	0.6907 (3)	0.4256 (3)	1.5345 (3)	0.0442 (7)	
N4	0.7120 (3)	0.5378 (3)	1.2194 (3)	0.0507 (7)	
C1	0.3741 (3)	0.1548 (3)	1.4219 (3)	0.0477 (9)	
C2	0.4614 (3)	0.2600 (3)	1.3994 (3)	0.0432 (8)	
C3	0.5805 (3)	0.3207 (3)	1.5104 (3)	0.0417 (8)	
C4	0.4568 (3)	0.3106 (3)	1.2988 (3)	0.0416 (8)	
C5	0.5739 (3)	0.4802 (4)	1.2173 (3)	0.0518 (9)	
H5A	0.5198	0.4087	1.1282	0.062*	
H5B	0.5338	0.5520	1.2358	0.062*	
C6	0.5706 (3)	0.4201 (3)	1.3199 (3)	0.0432 (8)	
C7	0.6845 (3)	0.4735 (3)	1.4386 (3)	0.0417 (8)	
C8	0.8071 (3)	0.5867 (3)	1.4584 (3)	0.0435 (8)	
C9	0.7958 (4)	0.6439 (4)	1.3528 (4)	0.0565 (10)	
H9A	0.7567	0.7160	1.3767	0.068*	
H9B	0.8861	0.6849	1.3509	0.068*	
C10	0.6506 (3)	0.2705 (3)	1.7162 (3)	0.0458 (8)	
C11	0.5969 (4)	0.2282 (4)	1.8042 (4)	0.0588 (10)	
H11	0.5032	0.1886	1.7824	0.071*	
C12	0.6838 (4)	0.2456 (4)	1.9247 (4)	0.0659 (11)	
H12	0.6477	0.2174	1.9836	0.079*	
C13	0.8214 (4)	0.3032 (4)	1.9589 (4)	0.0690 (11)	
H13	0.8788	0.3151	2.0405	0.083*	
C14	0.8741 (4)	0.3437 (4)	1.8704 (4)	0.0643 (11)	
H14	0.9679	0.3820	1.8923	0.077*	
C15	0.7896 (4)	0.3281 (4)	1.7496 (4)	0.0555 (10)	
H15	0.8265	0.3564	1.6911	0.067*	
C16	0.2334 (4)	0.0573 (4)	1.3364 (4)	0.0625 (11)	
H16A	0.2020	−0.0031	1.3773	0.094*	
H16B	0.2341	0.0036	1.2485	0.094*	
H16C	0.1737	0.1087	1.3284	0.094*	
C17	0.3367 (3)	0.2461 (3)	1.1741 (3)	0.0459 (8)	
C18	0.2173 (3)	0.2811 (4)	1.1483 (3)	0.0498 (9)	
H18	0.2016	0.3556	1.2057	0.060*	
C19	0.1251 (4)	0.1809 (5)	1.0180 (4)	0.0675 (11)	
H19	0.0395	0.1832	0.9821	0.081*	
C20	0.1702 (4)	0.0850 (4)	0.9523 (4)	0.0657 (11)	
H20	0.1202	0.0143	0.8675	0.079*	
C21	0.7112 (4)	0.5941 (4)	1.1182 (4)	0.0688 (11)	
H21A	0.6811	0.6719	1.1425	0.103*	
H21B	0.6506	0.5245	1.0320	0.103*	
H21C	0.8015	0.6230	1.1136	0.103*	
C22	0.9196 (3)	0.6288 (3)	1.5629 (3)	0.0482 (9)	
H22	0.9119	0.5826	1.6186	0.058*	
C23	1.0514 (4)	0.7338 (3)	1.6046 (3)	0.0480 (9)	
C24	1.1687 (3)	0.7475 (3)	1.7027 (3)	0.0425 (8)	
H24	1.1705	0.6932	1.7511	0.051*	
C25	1.2830 (4)	0.8555 (4)	1.7167 (4)	0.0665 (11)	
H25	1.3696	0.8787	1.7750	0.080*	
C26	1.2548 (4)	0.9214 (4)	1.6380 (4)	0.0740 (13)	
H26	1.3193	0.9945	1.6364	0.089*	

### Atomic displacement parameters (Å^2^)

**Table:**

	*U*^11^	*U*^22^	*U*^33^	*U*^12^	*U*^13^	*U*^23^
S1	0.0715 (8)	0.0687 (7)	0.0740 (8)	−0.0057 (6)	0.0132 (6)	0.0382 (6)
S2	0.0723 (8)	0.0782 (8)	0.0661 (7)	0.0296 (6)	0.0035 (6)	0.0026 (6)
N1	0.0430 (17)	0.0511 (17)	0.0446 (16)	0.0028 (14)	0.0117 (14)	0.0245 (14)
N2	0.0424 (17)	0.0533 (18)	0.0526 (18)	−0.0015 (14)	0.0116 (14)	0.0248 (15)
N3	0.0400 (16)	0.0432 (15)	0.0403 (15)	0.0046 (13)	0.0104 (13)	0.0154 (13)
N4	0.0521 (18)	0.0571 (17)	0.0439 (16)	0.0121 (15)	0.0142 (14)	0.0278 (15)
C1	0.042 (2)	0.047 (2)	0.048 (2)	0.0066 (16)	0.0138 (16)	0.0192 (17)
C2	0.0382 (19)	0.0431 (18)	0.0415 (18)	0.0080 (15)	0.0127 (15)	0.0140 (15)
C3	0.0405 (19)	0.0447 (18)	0.0362 (18)	0.0095 (16)	0.0102 (15)	0.0177 (15)
C4	0.0378 (19)	0.0428 (18)	0.0402 (18)	0.0126 (15)	0.0104 (15)	0.0147 (15)
C5	0.049 (2)	0.057 (2)	0.051 (2)	0.0140 (18)	0.0126 (17)	0.0300 (18)
C6	0.042 (2)	0.0457 (19)	0.0436 (19)	0.0134 (16)	0.0157 (16)	0.0204 (16)
C7	0.0414 (19)	0.0432 (18)	0.0415 (19)	0.0128 (16)	0.0150 (16)	0.0192 (16)
C8	0.046 (2)	0.0409 (18)	0.0402 (18)	0.0103 (16)	0.0160 (16)	0.0157 (15)
C9	0.056 (2)	0.056 (2)	0.057 (2)	0.0079 (19)	0.0181 (19)	0.0306 (19)
C10	0.047 (2)	0.0435 (19)	0.045 (2)	0.0110 (16)	0.0141 (17)	0.0204 (16)
C11	0.055 (2)	0.065 (2)	0.054 (2)	0.0109 (19)	0.0169 (19)	0.029 (2)
C12	0.075 (3)	0.080 (3)	0.054 (2)	0.025 (2)	0.026 (2)	0.040 (2)
C13	0.070 (3)	0.086 (3)	0.052 (2)	0.029 (2)	0.012 (2)	0.033 (2)
C14	0.050 (2)	0.072 (3)	0.063 (3)	0.017 (2)	0.008 (2)	0.027 (2)
C15	0.055 (2)	0.062 (2)	0.053 (2)	0.0169 (19)	0.0186 (19)	0.0284 (19)
C16	0.048 (2)	0.061 (2)	0.063 (2)	−0.0026 (19)	0.0112 (19)	0.027 (2)
C17	0.044 (2)	0.0469 (19)	0.0428 (19)	0.0086 (16)	0.0146 (16)	0.0186 (16)
C18	0.041 (2)	0.058 (2)	0.0372 (18)	0.0101 (17)	0.0098 (16)	0.0105 (17)
C19	0.047 (2)	0.089 (3)	0.072 (3)	0.026 (2)	0.014 (2)	0.043 (3)
C20	0.055 (2)	0.061 (2)	0.049 (2)	0.001 (2)	−0.0019 (19)	0.0084 (19)
C21	0.072 (3)	0.083 (3)	0.059 (2)	0.018 (2)	0.021 (2)	0.045 (2)
C22	0.051 (2)	0.0463 (19)	0.047 (2)	0.0076 (17)	0.0196 (18)	0.0229 (17)
C23	0.051 (2)	0.0436 (19)	0.0426 (19)	0.0065 (17)	0.0194 (17)	0.0146 (16)
C24	0.0415 (19)	0.0375 (17)	0.0444 (19)	0.0098 (15)	0.0167 (16)	0.0132 (15)
C25	0.047 (2)	0.072 (3)	0.061 (2)	0.011 (2)	0.017 (2)	0.011 (2)
C26	0.064 (3)	0.060 (2)	0.068 (3)	−0.014 (2)	0.025 (2)	0.016 (2)

### Geometric parameters (Å, °)

**Table:**

S1—C26	1.672 (5)	C11—C12	1.384 (5)
S1—C23	1.718 (4)	C11—H11	0.9300
S2—C20	1.689 (4)	C12—C13	1.363 (5)
S2—C17	1.708 (3)	C12—H12	0.9300
N1—C3	1.375 (4)	C13—C14	1.379 (6)
N1—N2	1.381 (3)	C13—H13	0.9300
N1—C10	1.424 (4)	C14—C15	1.381 (5)
N2—C1	1.314 (4)	C14—H14	0.9300
N3—C3	1.334 (4)	C15—H15	0.9300
N3—C7	1.338 (4)	C16—H16A	0.9600
N4—C5	1.451 (4)	C16—H16B	0.9600
N4—C9	1.455 (4)	C16—H16C	0.9600
N4—C21	1.458 (4)	C17—C18	1.423 (5)
C1—C2	1.428 (4)	C18—C19	1.438 (5)
C1—C16	1.493 (5)	C18—H18	0.9300
C2—C3	1.398 (4)	C19—C20	1.325 (5)
C2—C4	1.407 (4)	C19—H19	0.9300
C4—C6	1.398 (4)	C20—H20	0.9300
C4—C17	1.487 (4)	C21—H21A	0.9600
C5—C6	1.504 (5)	C21—H21B	0.9600
C5—H5A	0.9700	C21—H21C	0.9600
C5—H5B	0.9700	C22—C23	1.448 (4)
C6—C7	1.420 (4)	C22—H22	0.9300
C7—C8	1.486 (4)	C23—C24	1.417 (5)
C8—C22	1.336 (5)	C24—C25	1.417 (5)
C8—C9	1.508 (5)	C24—H24	0.9300
C9—H9A	0.9700	C25—C26	1.344 (6)
C9—H9B	0.9700	C25—H25	0.9300
C10—C15	1.379 (5)	C26—H26	0.9300
C10—C11	1.389 (5)		
			
C26—S1—C23	93.1 (2)	C13—C12—H12	119.3
C20—S2—C17	92.60 (19)	C11—C12—H12	119.3
C3—N1—N2	110.2 (3)	C12—C13—C14	118.9 (4)
C3—N1—C10	130.4 (3)	C12—C13—H13	120.5
N2—N1—C10	119.4 (3)	C14—C13—H13	120.5
C1—N2—N1	107.4 (3)	C13—C14—C15	121.0 (4)
C3—N3—C7	114.8 (3)	C13—C14—H14	119.5
C5—N4—C9	110.4 (3)	C15—C14—H14	119.5
C5—N4—C21	110.0 (3)	C10—C15—C14	119.8 (4)
C9—N4—C21	110.6 (3)	C10—C15—H15	120.1
N2—C1—C2	110.4 (3)	C14—C15—H15	120.1
N2—C1—C16	120.6 (3)	C1—C16—H16A	109.5
C2—C1—C16	129.0 (3)	C1—C16—H16B	109.5
C3—C2—C4	117.5 (3)	H16A—C16—H16B	109.5
C3—C2—C1	105.4 (3)	C1—C16—H16C	109.5
C4—C2—C1	137.1 (3)	H16A—C16—H16C	109.5
N3—C3—N1	126.3 (3)	H16B—C16—H16C	109.5
N3—C3—C2	127.1 (3)	C18—C17—C4	128.8 (3)
N1—C3—C2	106.6 (3)	C18—C17—S2	111.7 (2)
C6—C4—C2	117.1 (3)	C4—C17—S2	119.5 (3)
C6—C4—C17	122.2 (3)	C17—C18—C19	108.0 (3)
C2—C4—C17	120.6 (3)	C17—C18—H18	126.0
N4—C5—C6	111.5 (3)	C19—C18—H18	126.0
N4—C5—H5A	109.3	C20—C19—C18	115.7 (4)
C6—C5—H5A	109.3	C20—C19—H19	122.2
N4—C5—H5B	109.3	C18—C19—H19	122.2
C6—C5—H5B	109.3	C19—C20—S2	112.1 (3)
H5A—C5—H5B	108.0	C19—C20—H20	124.0
C4—C6—C7	119.5 (3)	S2—C20—H20	124.0
C4—C6—C5	120.5 (3)	N4—C21—H21A	109.5
C7—C6—C5	120.0 (3)	N4—C21—H21B	109.5
N3—C7—C6	124.0 (3)	H21A—C21—H21B	109.5
N3—C7—C8	116.7 (3)	N4—C21—H21C	109.5
C6—C7—C8	119.3 (3)	H21A—C21—H21C	109.5
C22—C8—C7	119.9 (3)	H21B—C21—H21C	109.5
C22—C8—C9	124.1 (3)	C8—C22—C23	131.3 (3)
C7—C8—C9	115.9 (3)	C8—C22—H22	114.4
N4—C9—C8	112.0 (3)	C23—C22—H22	114.4
N4—C9—H9A	109.2	C24—C23—C22	123.9 (3)
C8—C9—H9A	109.2	C24—C23—S1	109.9 (2)
N4—C9—H9B	109.2	C22—C23—S1	126.2 (3)
C8—C9—H9B	109.2	C23—C24—C25	110.4 (3)
H9A—C9—H9B	107.9	C23—C24—H24	124.8
C15—C10—C11	119.6 (3)	C25—C24—H24	124.8
C15—C10—N1	120.7 (3)	C26—C25—C24	114.0 (4)
C11—C10—N1	119.7 (3)	C26—C25—H25	123.0
C12—C11—C10	119.4 (4)	C24—C25—H25	123.0
C12—C11—H11	120.3	C25—C26—S1	112.5 (3)
C10—C11—H11	120.3	C25—C26—H26	123.7
C13—C12—C11	121.3 (4)	S1—C26—H26	123.7
			
C3—N1—N2—C1	0.9 (4)	C6—C7—C8—C9	4.5 (5)
C10—N1—N2—C1	−178.9 (3)	C5—N4—C9—C8	62.2 (4)
N1—N2—C1—C2	−0.7 (4)	C21—N4—C9—C8	−175.8 (3)
N1—N2—C1—C16	179.8 (3)	C22—C8—C9—N4	143.5 (3)
N2—C1—C2—C3	0.3 (4)	C7—C8—C9—N4	−34.4 (4)
C16—C1—C2—C3	179.7 (3)	C3—N1—C10—C15	−24.3 (6)
N2—C1—C2—C4	178.8 (4)	N2—N1—C10—C15	155.4 (3)
C16—C1—C2—C4	−1.8 (7)	C3—N1—C10—C11	156.4 (3)
C7—N3—C3—N1	179.3 (3)	N2—N1—C10—C11	−23.9 (5)
C7—N3—C3—C2	−1.6 (5)	C15—C10—C11—C12	0.5 (6)
N2—N1—C3—N3	178.5 (3)	N1—C10—C11—C12	179.8 (3)
C10—N1—C3—N3	−1.8 (6)	C10—C11—C12—C13	−0.1 (6)
N2—N1—C3—C2	−0.7 (4)	C11—C12—C13—C14	−0.5 (6)
C10—N1—C3—C2	179.0 (3)	C12—C13—C14—C15	0.8 (6)
C4—C2—C3—N3	2.2 (5)	C11—C10—C15—C14	−0.2 (6)
C1—C2—C3—N3	−178.9 (3)	N1—C10—C15—C14	−179.5 (3)
C4—C2—C3—N1	−178.6 (3)	C13—C14—C15—C10	−0.4 (6)
C1—C2—C3—N1	0.3 (4)	C6—C4—C17—C18	−90.7 (4)
C3—C2—C4—C6	−1.7 (5)	C2—C4—C17—C18	91.2 (4)
C1—C2—C4—C6	180.0 (4)	C6—C4—C17—S2	92.1 (4)
C3—C2—C4—C17	176.6 (3)	C2—C4—C17—S2	−86.1 (4)
C1—C2—C4—C17	−1.8 (6)	C20—S2—C17—C18	−2.6 (3)
C9—N4—C5—C6	−58.1 (4)	C20—S2—C17—C4	175.1 (3)
C21—N4—C5—C6	179.6 (3)	C4—C17—C18—C19	−174.7 (3)
C2—C4—C6—C7	0.8 (5)	S2—C17—C18—C19	2.8 (4)
C17—C4—C6—C7	−177.4 (3)	C17—C18—C19—C20	−1.6 (5)
C2—C4—C6—C5	−180.0 (3)	C18—C19—C20—S2	−0.4 (5)
C17—C4—C6—C5	1.8 (5)	C17—S2—C20—C19	1.7 (3)
N4—C5—C6—C4	−151.4 (3)	C7—C8—C22—C23	179.2 (3)
N4—C5—C6—C7	27.8 (4)	C9—C8—C22—C23	1.3 (6)
C3—N3—C7—C6	0.6 (5)	C8—C22—C23—C24	−166.9 (4)
C3—N3—C7—C8	−177.7 (3)	C8—C22—C23—S1	12.1 (6)
C4—C6—C7—N3	−0.3 (5)	C26—S1—C23—C24	1.5 (3)
C5—C6—C7—N3	−179.5 (3)	C26—S1—C23—C22	−177.6 (3)
C4—C6—C7—C8	177.9 (3)	C22—C23—C24—C25	177.2 (3)
C5—C6—C7—C8	−1.3 (5)	S1—C23—C24—C25	−1.9 (3)
N3—C7—C8—C22	4.8 (5)	C23—C24—C25—C26	1.4 (4)
C6—C7—C8—C22	−173.6 (3)	C24—C25—C26—S1	−0.2 (5)
N3—C7—C8—C9	−177.2 (3)	C23—S1—C26—C25	−0.8 (3)

### Hydrogen-bond geometry (Å, °)

**Table:**

*D*—H···*A*	*D*—H	H···*A*	*D*···*A*	*D*—H···*A*
C26—H26···N2^i^	0.93	2.57	3.445 (3)	157
C20—H20···Cg6^ii^	0.93	2.93	3.680 (3)	139

Symmetry codes: (i) *x*+1, *y*+1, *z*; (ii) *x*+1, *y*+1, *z*+1.
